# Stem cell therapy as a promising strategy in necrotizing enterocolitis

**DOI:** 10.1186/s10020-022-00536-y

**Published:** 2022-09-06

**Authors:** Si-Jia Di, Si-Yuan Wu, Tian-Jing Liu, Yong-Yan Shi

**Affiliations:** Department of Pediatrics, Shengjing Hospital of China Medical University, Shenyang, 110004 China

**Keywords:** Necrotizing enterocolitis, Stem cells, Infants

## Abstract

Necrotizing enterocolitis (NEC) is a devastating gastrointestinal disease that affects newborns, particularly preterm infants, and is associated with high morbidity and mortality. No effective therapeutic strategies to decrease the incidence and severity of NEC have been developed to date. Stem cell therapy has been explored and even applied in various diseases, including gastrointestinal disorders. Animal studies on stem cell therapy have made great progress, and the anti-inflammatory, anti-apoptotic, and intestinal barrier enhancing effects of stem cells may be protective against NEC clinically. In this review, we discuss the therapeutic mechanisms through which stem cells may function in the treatment of NEC.

## Introduction

Despite constant efforts on improving the diagnosis and treatment technique of premature infants, the morbidity and mortality rates associated with necrotizing enterocolitis (NEC), a devastating gastrointestinal inflammatory and necrotizing disease that affects newborns, particularly preterm infants, are rising. As a main cause of death in the neonatal intensive care unit, NEC has an incidence of approximately 8.9% (890/9956) in premature infants born at the gestational age of 22–28 weeks, and the mortality rate associated with NEC can be as high as 20–30%. Infants requiring surgery exhibit higher mortality rates (Bell et al. 2022; Meister et al. 2020). Survivors may suffer from lifelong gastrointestinal problems, including strictures, adhesions, cholestasis, short bowel syndrome with or without intestinal failure, and neurological sequelae (Bazacliu and Neu 2019).

NEC develops in response to hypoxic-ischemic injury of the intestinal mucosa, caused by exaggerated pro-inflammatory signals and compromised anti-inflammatory signals (Cho et al. 2020). As the main mediator regulating the balance between mucosal injury and repair in the intestines of premature infants, Toll-like receptor 4 is upregulated in infants with NEC, and inactivation of this protein has protective effects in stem cells (Hackam and Sodhi 2018; Liu et al. 2019; Niu et al. 2018). Because NEC is a multifactorial disorder, few radical treatments have been developed, so nonspecific supportive care based on diagnosis and surgery is the primary therapeutic approach. However, stem cell therapy has recently been evaluated in the treatment of NEC owing to the self-renewal potential, multidirectional differentiation capacity, good availability of stem cells, as well as their effects on protecting the intestinal barrier, inhibiting apoptosis, and reducing inflammation (Sajeesh et al. 2020; Pisano and Besner 2019).

In this review, we discuss current progress of stem cell therapy in NEC, with the goal of further elucidating the therapeutic mechanisms of stem cells in NEC and promoting breakthroughs in clinical trials. Accordingly, our review provides potential insight for the progress of new therapeutic method for NEC.

## Stem cells

Stem cells are a class of unspecialized or undifferentiated cells that can self-renew and produce highly differentiated mature daughter cells. Stem cells can be divided into totipotent, pluripotent, and unipotent stem cells according to their differentiation potential. Totipotent cells, such as zygotes, have the potential for multidirectional differentiation; pluripotent stem cells, including embryonic stem cells (ESCs), can differentiate into multiple tissues, but cannot develop into complete individuals; and unipotent stem cells, including neural stem cells (NSCs), refer to cells that can only differentiate into one type of cell. Additionally, stem cells are classified into ESCs, adult stem cells (ASCs) and induced pluripotent stem cells (iPSCs), a novel type of stem cell identified in recent years according to developmental origins (Bozdağ et al. 2018).

**Fig. 1:**
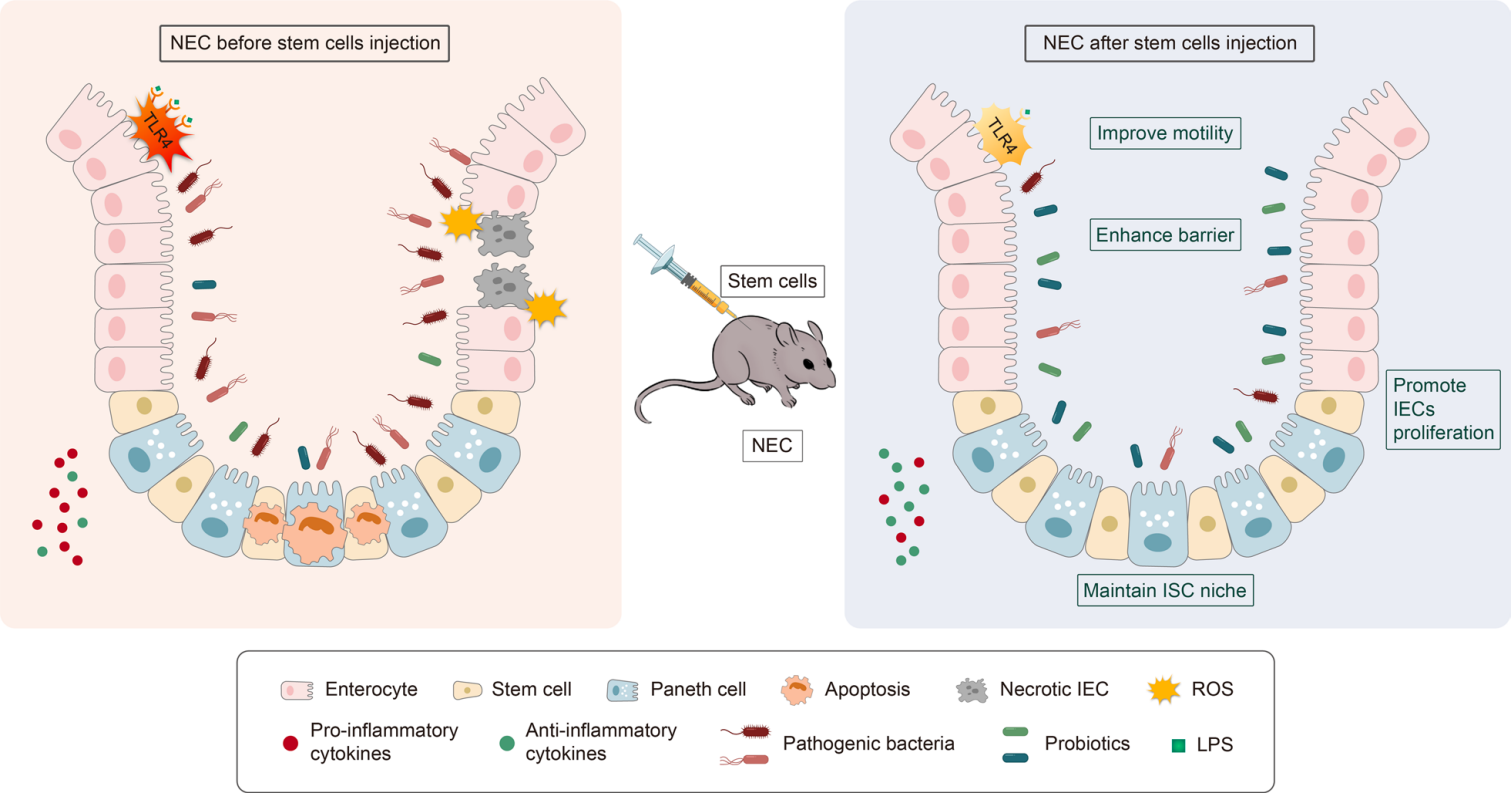
Therapeutic effects of stem cells in NEC. Schematic illustrating the therapeutic effects of stem cells in the injured intestines of NEC model rats. With the injection of stem cells, the experimental NEC intestine has been shown to decrease inflammation, apoptosis, necrosis, and oxidant stress, balance bacteria, enhance barrier, inactivate TLR4, maintain ISC niche, improve motility, and promote IECs proliferation. *ROS* reactive oxygen species; *LPS* lipopolysaccharide; *TLR4* toll-like receptor 4

ASCs are pluripotent stem cells that are commonly used in the clinical setting. These cells can proliferate and differentiate directionally into certain unipotent stem cells, such as hematopoietic stem cells (HSCs), mesenchymal stem cells (MSCs), and intestinal stem cells (ISCs) (Suman et al. 2019). Such stem cells have beneficial functions in the intestine, including promoting intestinal epithelium growth, regulating inflammatory cytokines, reducing cell apoptosis, decreasing oxidant stress, repairing the intestinal barrier, and so on (Kandasamy et al. 2014; Jung et al. 2020; Hou et al. 2017; Burns and Thapar 2014) (Fig. 1).

## Stem cell therapy in NEC

Considering the indispensable roles of stem cells in intestinal protection, stem cell therapy has attracted much interest in studies of NEC. A meta-analysis including nine animal experiments suggested that stem cells and stem cell-derived exosomes decreased the morbidity of NEC, particularly stage 2 NEC by enhancing intestinal motility and reducing intestinal permeability (Villamor-Martinez et al. 2020; Walsh et al. 1988) (Table 1). Current research indicates that ISCs, MSCs, and NSCs, which are derived from various tissues, are most commonly used for NEC treatment (Li et al. 2022; Zhou et al. 2013; Zeng et al. 2021; Drucker et al. 2018a). In the subsequent sections, we discuss how stem cells treat NEC as well as their advantages and disadvantages in the clinical setting (Table 2).

**Table 1:** Modified Bell’s staging criteria for NEC

Stage	Systemic signs	Intestinal signs
I (Suspected)	Temperature instability, apnea, bradycardia	Elevated pregavage residuals, mild abdominal distention, occult blood in stool
II (Definite)	Same as I, plus mild metabolic acidosis, mild thrombocytopenia	Same as above, plus absent bowel sounds, definite abdominal tenderness, abdominal cellulitis, right lower quadrant mass
III (Advanced)	Same as I, plus hypotension, bradycardia, respiratory acidosis, metabolic acidosis, disseminated intravascular coagulation, neutropenia	Same as above, plus signs of generalized peritonitis, marked tenderness, and distention of abdomen

**Table 2:** Main studies on stem cell therapy in NEC

Stem cell type (location)	Main mechanisms	Benefits	Limitations	References
ISCs (Base of intestinal crypts)	Paracrine; other signaling pathways	Regulate intestinal microbiota, mucosal immune response, inflammatory cytokines and cell apoptosis	Over-reliance on the balance between proliferation and differentiation	Kandasamy et al. (2014)
BM-MSCs (Bone marrow)	Paracrine trophic factors; prolyl hydroxylase 2 silencing	Notably decrease inflammation; improve tissue pathology	Low proliferative ability; effect is affected by donor age	McCulloh et al. (2017b), Weil et al. (2009), Chen et al. (2020), Alves et al. (2012), Kagia et al. (2019), Li et al. (2018)
AF-MSCs (Amniotic fluid)	COX-2; Wnt; ER stress	Easy to cultivate; enhance clinical translation; prevent ascites; prolong survival; low tumorigenicity	Few clinical trials to assess the safety	McCulloh et al. (2017b), Jensen et al. (2016), Rosner and Hengstschläger (2021), Zani et al. (2014b)
UC-MSCs (Umbilical cord/umbilical cord blood)	Nitric oxide synthase; hydrogen sulfide	Notably decrease inflammation; improve tissue pathology	Uncertainty of abnormal long-term development	McCulloh et al. (2017b), Jensen et al. (2018), Drucker et al. (2019), Kagia et al. (2019), Prather et al. (2008)
P-MSCs (Placenta)	Wnt; paracrine effects	Good availability; few ethical problems	Few clinical trials to assess the safety	Weis et al. (2021), Duan et al. (2020), Khalifeh Soltani et al. (2019)
NSCs (Intestine/amniotic fluid)	ENS; neuronal nitric oxide synthase	Reduce immunological rejection by autologous transplantation; improve intestinal motility	Difficult to separate and cultivate; ineffective IP injection; little improvement in inflammation	Burns and Thapar (2014), McCulloh et al. (2017b), Kagia et al. (2019), Nitkin et al. (2020)

*ISCs* intestinal stem cells; *MSCs* mesenchymal stem cells; *BM* bone marrow; *AF* amniotic fluid; *COX-2* cyclooxygenase-2; *ER* endoplasmic reticulum; *UC* umbilical cord; *P* placenta; *NSCs* neural stem cells; *ENS* enteric nervous system; *IP* intraperitoneal

### ISCs

Disruption of the viability and integrity of the intestinal epithelium and injury to the intestinal mucosa are commonly observed in infants with NEC (Yu et al. 2020). As a rapidly renewing tissue in mammals, the intestinal epithelium is mainly differentiated from ISCs located at the base of crypts. Thus, ISCs may be involved in the development of NEC (Venkatraman et al. 2021; Neal et al. 2012).

Two types of ISCs are present in intestinal crypts: active ISCs (actively proliferating) and reserve or quiescent ISCs (quiescent cycling). Active ISCs, which can be identified by the marker leucine-rich repeat-containing G protein-coupled receptor 5 (LGR5), are responsible for promoting homeostatic renewal and differentiation to intestinal epithelial cells (IECs) (Stewart et al. 2021). The regeneration of small intestinal crypts and villi is mainly attributed to the colonization of reserve ISCs. Reserve ISCs transform into active ISCs by silencing homeodomain-only protein X and active ISCs, then migrate to the damaged segment of the intestine and play important roles in injury-induced intestinal regeneration (Stewart et al. 2021; Gonzalez et al. 2019).

Goblet cells (Zhao et al. 2021), Paneth cells (Barreto et al. 2022), and enteroendocrine cells (Landeghem et al. 2012) derived from ISCs are crucial in modulating the proliferation and differentiation of IECs and promoting intestinal development via the paracrine signaling pathways, including the hedgehog, BMP, Wnt/β-catenin, and Notch signaling pathways; other pathways, such as endocrine signaling pathways and transcription factor pathways, also play important roles (Venkatraman et al. 2021). Owing to their roles in regulating the intestinal microbiota, mucosal immune responses, inflammatory cytokines, and cell apoptosis, paracrine pathways may protect infants from NEC.

### MSCs

MSCs are ASCs that originate from the mesoderm and can differentiate into various mesenchymal cells, depending on the tissue in which they are located, e.g., the bone marrow, amniotic fluid, umbilical cord, placental tissues, dental pulp, and adipose tissue (Zhan et al. 2019). MSCs are the first type of stem cell studied in detail and are characterized by proliferation in vitro, multipotency, homing/migration, trophic effects, and immunosuppression; thus, these cells have been shown to have therapeutic potential in multiple autoimmune, inflammatory, and degenerative diseases (Naji et al. 2019). Moreover, MSCs have been shown to reduce the incidence and severity of experimental NEC in rats, although the mechanisms are still unclear (McCulloh et al. 2017a, b).

Notably, MSCs can home to injured intestinal segments; however, the number of MSCs observed in the intestine is not sufficient to exert protective effects, indicating that this protective action may be mainly related to another mechanism (Bahr et al. 2012). Additionally, the efficacy of MSCs may be predominantly mediated by paracrine chemokines and/or growth factors, e.g., interleukin (IL)-6 (Gu et al. 2022), IL-10 (Tu et al. 2022), vascular endothelial growth factor (Chou et al. 2016), and transforming growth factor-β (TGF-β) (Barati et al. 2022). IL-6 inhibits apoptosis, IL-10 exerts anti-inflammatory effects, vascular endothelial growth factor plays important roles in angiogenesis, and TGF-β blocks the expression of pro-inflammatory factors (Gu et al. 2022; Tu et al. 2022; Chou et al. 2016; Barati et al. 2022). These growth factors migrate to the ischemic intestine tissue and contribute to the treatment of NEC. It is worth noting that the paracrine effect of MSCs seems to be mediated through a “hit and run” mechanism. This short-acting and transient engraftment in the injured intestine may limit the adverse effects of MSCs therapy (Bahr et al. 2012).

The anti-inflammatory, antioxidant, anti-apoptotic, and local LGR5^+^ ISCs proliferative effects of MSCs, coupled with their effects on enhancement of gut microbial diversity in a colitis rat model could synergistically facilitate intestinal recovery (Jung et al. 2020; Weil et al. 2009; Soontararak et al. 2018). Moreover, regardless of the origin of tissue, transplantation of MSCs after intestinal ischemia/reperfusion prominently increases survival rates, reverses mesenteric perfusion, and blocks intestinal injury and inflammation (Jensen et al. 2016).

### Bone marrow-derived MSCs (BM-MSCs)

As the name suggests, BM-MSCs are MSCs that originated from the bone marrow. Experiments evaluating the therapeutic effects of exogenous human BM-MSCs in a neonatal rat NEC model demonstrated that after intraperitoneal (IP) injection of transplanted BM-MSCs, the concentration of exogenous human BM-MSCs was increased in the area of the injured intestinal segment, with amelioration of intestinal pathological damage (Tayman et al. 2011).

The functions of BM-MSCs are mainly mediated by paracrine factors. Importantly, inhibition of the upstream transcription factor prolyl hydroxylase 2 enhances the paracrine efficacy of BM-MSCs and protects against NEC. The reason for this phenomenon is that prolyl hydroxylase 2 silencing promotes nuclear factor-κB activation to increase the release of the protective factors—insulin-like growth factor-1 and TGF-β2. Moreover, deficiency in prolyl hydroxylase 2 increases survival in NEC by modulating epithelial regeneration and inflammatory responses (Chen et al. 2020).

### Amniotic fluid-derived MSCs (AF-MSCs)

AF-MSCs are a subset of MSCs extracted from amniotic fluid. These cells exhibit rapid proliferation and multidirectional differentiation, similar to pluripotent stem cells (Kaviani et al. 2001). AF-MSCs are easier to harvest and expand in vitro than ASCs and differentiate into cell lineages of all three embryonic germ layers (Dasgupta and Jain 2017; Rosner and Hengstschläger 2021). Additionally, AF-MSCs transiently stimulate healthy IECs proliferation and preserve LGR5^+^ ISCs, regardless of intestinal injury; thus, these cells may represent novel therapeutic agents in NEC (Li et al. 2022). Moreover, AF-MSCs have recently been shown to reduce the incidence and severity of NEC (Stenson 2014; Zani et al. 2014a; Li et al. 2020).

Wnt signaling is crucial for maintaining ISCs and IECs homeostasis, whose impairment has been observed in experimental NEC model rats (Li et al. 2019). Moreover, AF-MSCs have been shown to activate Wnt signaling, leading to the rescue of injured ISCs, decreased apoptosis and mucosal inflammation, proliferation of IECs, and restoration of intestinal construction (Li et al. 2020).

In addition, AF-MSCs rely on endoplasmic reticulum (ER) stress to mediate NEC. AF-MSCs activate the ER stress response to process the unfolded tight junction proteins, such as claudin-7, which can influence the function of the intestinal barrier by decreasing intestinal permeability (Li et al. 2021a). Meanwhile, AF-MSCs antagonize the apoptotic effects of ER stress by activating the binding immunoglobulin protein (ER stress central regulatory protein) and upregulating C/EBP homologous protein (modulator of apoptosis gene expression). These substances can further inhibit the expression of the pro-apoptotic marker, Bax, and stimulate the expression of the anti-apoptotic marker, Bcl-2, thereby inhibiting the necrosis and apoptosis of IECs (Li et al. 2021a; Lau et al. 2021).

IP injection of AF-MSCs was shown to reduce morbidity and mortality rates in NEC and prolong survival rates by increasing the expression of cyclooxygenase 2 (COX-2) in the lamina propria (Zani et al. 2014a). COX-2 is an important enzyme, whose expression is inversely proportional to the severity of NEC at 24 h (Lu and Zhu 2014). AF-MSCs secrete growth factors, which activate COX-2 either directly or indirectly by promoting the activation of epidermal growth factor receptors, thereby suppressing gut oxidation, facilitating villus cells proliferation, and reducing apoptosis (Zani et al. 2014a). Moreover, AF-MSC-mediated activation of COX-2 results in the secretion of tumor necrosis factor-induced protein 6, which can migrate to injured ileum tissue and attenuate intestinal ischemia/reperfusion injury, thereby blocking the onset of NEC (Koike et al. 2020; Klinke et al. 2020).

### Other types of MSCs

The efficacy of stem cells may be negatively correlated with donor age; therefore, identification of original progenitor cell sources, such as umbilical cord blood or placental tissue, is essential for the collection of alternatives to traditional BM-MSCs (Alves et al. 2012). Umbilical cord-derived stem cells (UC-MSCs) have the same pluripotency as BM-MSCs and greater anti-inflammatory and immunomodulatory potential than BM-MSCs (Wegmeyer et al. 2013). Additionally, these cells can be easily isolated using noninvasive methods. Placental-derived MSCs (P-MSCs), initially regarded as medical waste after delivery, have also been shown to be a good source of abundant stem cells with low immunogenicity and strong anti-inflammatory effects (Damianos et al. 2022).

As prenatal stem cells, the effects of UC-MSCs and P-MSCs on amelioration of intestinal damage are primarily mediated by paracrine signaling. Indeed, the administration of UC-MSCs enhances mesenteric perfusion, maintains the intestinal barrier, increases the expression of anti-inflammatory cytokines, and decreases the expression of pro-inflammatory cytokines owing to activation of endothelial nitric oxide synthase (Jensen et al. 2018). The effects of UC-MSCs on inhibition of intestinal damage by secreting hydrogen sulfide suggest potential applications in NEC therapy (Drucker et al. 2019, 2018b). Additionally, significant restoration of the ISC niche with increased Wnt/β-catenin signaling is crucial for the efficacy of human P-MSCs therapy in NEC (Weis et al. 2021). The paracrine effects of P-MSCs can also attenuate inflammation, promote mucosal recovery, and inhibit oxidative stress (Duan et al. 2020). Collectively, human P-MSCs can halt the progression of NEC-related damage to the intestine by improving epithelial morphology and inhibiting intestinal destruction.

### NSCs

The enteric neural system (ENS) refers to the multipotent cell population developing from enteric neural crest cells, which proliferate and differentiate into enteric neurons and glia cells in the developing intestine (Nagy and Goldstein 2017). ENS directly regulates gastrointestinal motility and secretes neurotransmitters to maintain intestinal mucosal epithelial barrier function independent from the central nervous system. The loss of enteric neurons and glia cells exacerbates the inflammatory cascades leading to intestinal ischemia (Nezami and Srinivasan 2010).

Both the immature ENS in premature infants and pathologically absent neurons and glial cells in the ENS leave infants susceptible to inflammatory injury observed in NEC (Chandramowlishwaran et al. 2022). Meanwhile, NEC epithelial injury also can cause ENS damage, and thus a vicious loop forms (Bellodas Sanchez and Kadrofske 2019). NSCs are responsible for repairing and replenishing neurons in the ENS, and their engraftment leads to enhancement of intestinal motility and prolongation of survival in experimental NEC (Burns and Thapar 2014; Zhou et al. 2013). Moreover, enteric NSCs (E-NSCs) significantly activate the neuronal nitric oxide synthase and increase nitric oxide, thereby preventing the ENS from damage and preserving intestinal integrity (Zhou et al. 2017).

However, it is difficult to extract NSCs from the gut, and researchers have instead focused on identifying convenient sources, such as the amniotic fluid. NSCs isolated from the amniotic fluid (AF-NSCs) also reduce the incidence and severity of NEC, and their protective effects have been shown to be equivalent to those of E-NSCs (Pisano and Besner 2019). Moreover, AF-NSCs can be collected at delivery or through amniocentesis and are more easily expanded in culture than E-NSCs (Coppi et al. 2007).

**Fig. 2:**
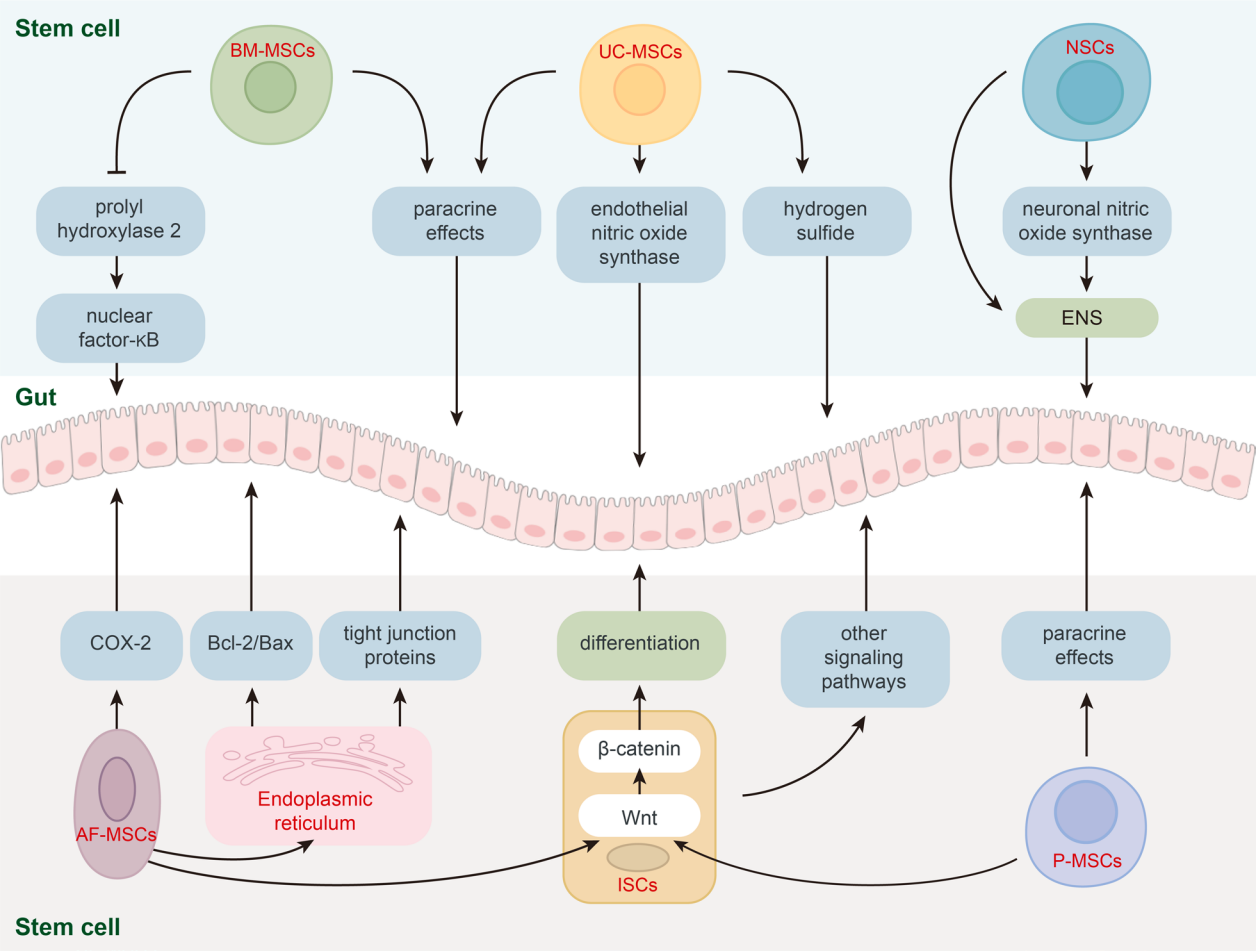
The different types of stem cells used to treat NEC and their signaling pathways. Stem cells exert NEC therapeutic effects via various signaling pathways. Mainly, MSCs exert therapeutic effects through paracrine signaling. In addition, BM-MSCs inhibit prolyl hydroxylase 2 to promote nuclear factor-κB activation and increase the release of the intestinal protective factors. UC-MSCs activate endothelial nitric oxide synthase and secrete hydrogen sulfide in NEC therapy. NSCs can repair and replenish neurons in the ENS, activate the neuronal nitric oxide synthase to prevent the ENS from being damaged, and preserve intestinal integrity. AF-MSCs activate ER stress response to process the unfolded tight junction proteins and promote the expression of Bcl-2/Bax gene. Moreover, AF-MSCs increase the expression of COX-2 in the lamina propria. AF-MSCs and P-MSCs restore the ISC niche to promote IECs proliferation with increased Wnt/β-catenin signaling. ISCs are deservedly responsible for the differentiation to IECs via various signaling pathways. *BM-MSCs* bone marrow-derived mesenchymal stem cells; *UC-MSCs* umbilical cord-derived stem cells; *NSCs* neural stem cells; *ENS* enteric neural system; *COX-2* cyclooxygenase 2; *Bcl-2* B-cell lymphoma 2; *Bax* Bcl-2-associated X protein; *AF-MSCs* amniotic fluid-derived mesenchymal stem cells; *ISCs* intestinal stem cells; *P-MSCs* placental-derived mesenchymal stem cells

Accordingly, stem cells can exert intestinal protective functions through diverse mechanisms (Fig. 2); however, much more work is necessary to fully elucidate these mechanisms. In addition to the therapeutic mechanisms of stem cells described above, previously published studies have also shown that several interventions can affect NEC by enhancing or hampering the therapeutic effects of stem cells (Table 3).

**Table 3:** Factors influencing stem cell therapy in NEC

Stem cell type	Intervention	Protection or risk	Key mechanisms	References
ISCs	HB-EGF	Protection	Protect ISCs from injury by PI3K and EGFR/MEK1/2/ERK1/2 pathways	Chen et al. (2012)
ISCs	Retinoic acid	Protection	Prevent apoptosis; protect ISCs by balancing pro-inflammatory Th17 and anti-inflammatory Tregs	Nino et al. (2017)
ISCs	Exosomes from human milk	Protection	Protect ISCs from oxidative stress injury through Wnt/β-catenin signaling	Dong et al. (2020)
ISCs	Corticotropin-releasing hormone receptor 2	Protection	Enhance ISCs expression via phosphorylation of STAT3 and IL-22	Li et al. (2017)
ISCs	Combination of multiple stress factors	Risk	Diminish expression of LGR5^+^ ISCs	Lee et al. (2018)
BM-MSCs	HB-EGF	Protection	Reduce apoptosis; promote migration and proliferation; facilitate MSCs engraftment and protect engrafted MSCs	Yang et al. (2012a)
AF-MSCs	HB-EGF	Protection	Increase chemotaxis; protect AF-MSCs against hypoxia-induced apoptosis effectively	Watkins et al. (2012)
NSCs	HB-EGF	Protection	Elevate enteric neuronal nitric oxide synthase levels; promote differentiation, migration, and proliferation of NSCs by epidermal growth factor receptor	Zhou et al. (2017), Shelby et al. (2019), Wei et al. (2015)

*ISCs* intestinal stem cells; *HB-EGF* heparin-binding epidermal growth factors; *PI3K* phosphatidylinositol 3-kinase; *STAT3* signal transducer and activator of transcription 3; *IL* interleukin; *LGR5* leucine-rich repeat-containing G protein-coupled receptor 5; *BM* bone marrow; *MSCs* mesenchymal stem cells; *AF* amniotic fluid; *NSCs* neural stem cells

## Applications of stem cells in NEC

Many advances have been made in the use of stem cell therapy in regeneration medicine in recent decades. Although stem cell-related research in NEC is limited, the feasibility of this approach is high. Further acceleration of progression in NEC treatment will require the removal of obstacles to stem cell transplantation.

### Origins of stem cells

**Fig. 3:**
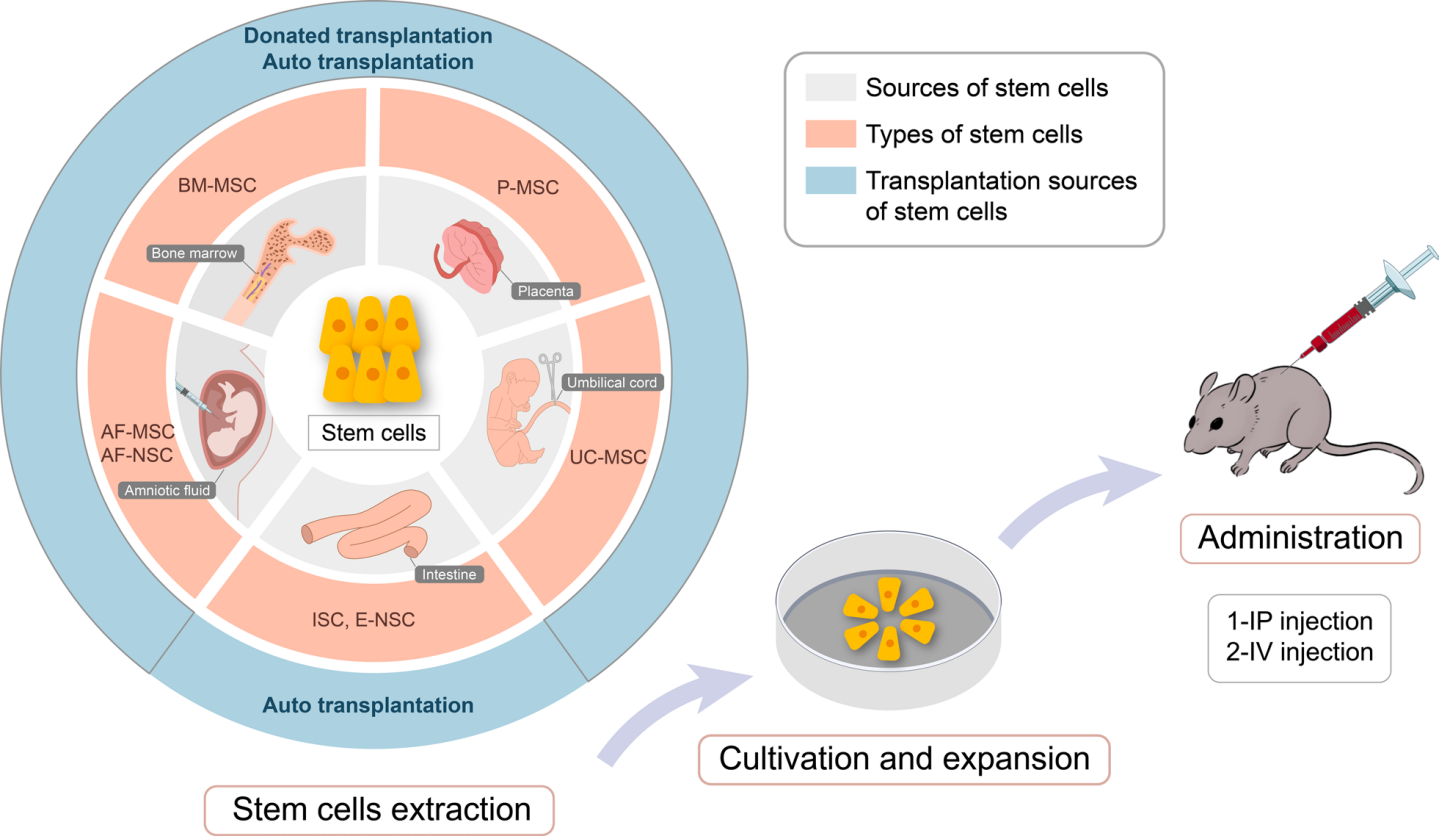
Stem cells from various sources are transplanted into NEC models. First, stem cells are isolated and extracted from various tissues including the intestine, bone marrow, amniotic fluid, umbilical cord, and placenta. Among, ISC and E-NSC are autologous, BM-MSC, AF-MSC, UC-MSC, P-MSC, and AF-NSC can be administered into NEC animal models by the donor or autologous transplantation. Then, extracted stem cells are propagated in an incubator. Finally, these cultured stem cells will be transported into NEC animal models via IP or IV injection. *BM-MSC* bone marrow-derived mesenchymal stem cell; *UC-MSC* umbilical cord-derived stem cell; *AF-MSC* amniotic fluid-derived mesenchymal stem cell; *AF-NSC* neural stem cell isolated from the amniotic fluid; *E-NSC* enteric neural stem cell; *ISC* Intestinal stem cell; *P-MSC* placental-derived mesenchymal stem cell; *IP* intraperitoneal; *IV* intravenous

MSCs that are derived from bone marrow, amniotic fluid, umbilical cord, and placenta have the great properties of low immunogenicity and immunosuppression, so these stem cells can be transplanted into NEC infants not only by autologous but by allogenic donor transplantation (Naji et al. 2019). AF-MSCs, AF-NSCs, UC-MSCs, and P-MSCs with low expression of human leukocyte antigen (HLA) lower the risk of rejection in allogeneic transplantation and can be easily collected at delivery (Gorodetsky and Aicher 2021). ISCs and E-NSCs are extracted from healthy regions of the patients for autologous transplantation, which avoids the issue of HLA matching in HSCs engraftment (Fig. 3).

### Approach of transplantation

Classical stem cell transplantation involves either IP or intravenous (IV) injection of cells (Ramalho et al. 2018). No major differences in morbidity, pathological damage, or survival rate were observed when comparing IP and IV administration of MSCs, although IV administration is more convenient in preclinical and clinical trials and is regarded as a more efficient delivery route than IP injection (Yang et al. 2012a, b). Nevertheless, IP injection prevents retention of transplanted cells in the pulmonary capillary, which is observed after IV injection, and avoids embolism when administered intra-arterially. IP injection also results in diffuse implantation throughout the gastrointestinal tract, particularly when a broad area of the intestine is treated (Nikiforou et al. 2016; Furlani et al. 2009). Researchers have also shown that systemic delivery of MSCs by umbilical vein infusion, which is safe, noninvasive, and effective, has a high success rate and is related to a low death rate (Yang et al. 2012b, 2014). Each type of injection has specific advantages, and the appropriate approach should be chosen according to a patient’s individual characteristics.

### Time of transplantation

It is still ambiguous whether the effects of stem cells on NEC are preventive or protective, which may affect the delivery time of stem cells (Eaton et al. 2013). Although the early use of stem cells is effective, prophylactic administration of stem cells in infants may raise ethical issues, and the treatment time window cannot be identified for therapeutic administration. Hence, it may be necessary to further optimize effective detection methods for NEC to facilitate early intervention with stem cell therapy.

### Clinical therapy

Because of the roles of stem cells in experimental NEC models, stem cell therapy was successfully applied in a clinical case in 2019. A 26-day-old full-term infant suffering from NEC was provided with IV injection of UC-MSCs after surgery, and mesenteric blood supply was significantly improved, revealing the potential of stem cells in NEC therapy and preventing short bowel syndrome in this infant (Akduman et al. 2021). However, this is the only published case of the clinical application of stem cells in NEC, and few clinical trials are currently being performed; indeed, on ClinicalTrials.gov, there is only one registered clinical trial for stem cell therapy in NEC to date (trial no. NCT05138276).

### Complications

Clinical transformation of stem cells is associated with multiple challenges, including ethical considerations, technical limitations, and adverse effects. Following transplantation, stem cells can exhibit abnormal differentiation after ectopic engraftment (Fennema et al. 2018), low survival rate (Reekmans et al. 2012), and can be physically trapped in the pulmonary capillary bed owing to the large diameter of the cells (Watanabe et al. 2019). Research has shown that undifferentiated stem cells can lead to teratoma formation in vivo (Li et al. 2021b). Therefore, stem cells with rapid growth and high differentiation capacity, such as UC-MSCs, may avoid tumorigenesis, immune rejection, and ethical problems, and are more suitable for current cell therapy approaches (Wang et al. 2022).

## Other stem cells derivatives

The conditioned medium is a mixture of all organic and inorganic products secreted by stem cells and can exert functions similar to those of stem cells. Extracellular vesicles (EVs) are vesicles coated with lipids, proteins, and RNA secreted from parental stem cells. The paracrine substances secreted by MSCs can exert function in other organs and even other individuals through the fusion with EVs, which is promising and sustainable for future NEC therapy (Joo et al. 2020). The transition from cell therapy to cell-free therapy may broaden the availability and safety of stem cell treatment.

Given that paracrine mediators are collected in conditioned medium from MSC (MSC-CM), MSC-CM could contribute to mucosal recovery, reduce inflammation, and restore ISCs activity (Lykov et al. 2018; O'Connell et al. 2021). Thus, MSC-CM may have applications in NEC. Owing to problems with low grafting efficiency caused by deficiencies in trophic paracrine factors in MSC-CM, it is also necessary to evaluate how to enhance the secretion of trophic paracrine factors and elevate the therapeutic efficacy of MSC-CM. Stimulation with several factors, including hypoxia, cytokines, growth factors, hormones, and drugs can yield an MSC-CM with protective effects against NEC in the intestinal tract of newborn rats, regulate the balance of pro-inflammatory factors and anti-inflammatory factors, and thereby reduce intestinal injury (Ferreira et al. 2018).

As a subpopulation of EVs, exosomes do not induce immunoreactions between HLA and stem cells owing to intercellular fusion. Moreover, these vesicles exhibit lower immunogenicity than stem cells (Manchon et al. 2021). Additionally, exosomes can cross the blood–brain barrier, enabling applications in brain injury caused by neurological sequelae related to NEC. Exosomes can also be used as delivery vehicles, e.g., facilitating the transportation of intestinal protective growth factors to the damaged intestine (Ghosh et al. 2020; McCulloh et al. 2018). Although exosomes can overcome many drawbacks of stem cell therapy, standardized production of large amounts of exosomes has not yet been achieved, and the legal and ethical considerations have not yet been discussed (Watanabe et al. 2021).

iPSCs have the potential for multi-dermal differentiation, the excellent ability of living and infinite progeny MSCs generation (Hynes et al. 2018). Importantly, they are derived from human’s cells and the differentiation ability can be induced in vitro from those adult cells after birth (Suman et al. 2019; Zakrzewski et al. 2019). There is no immune rejection, and the tumorigenicity evaluation of implanting differentiated MSCs in non-human primates didn’t show any evidence of tumor formation (Hong et al. 2014). Mouse iPSCs-derived MSCs have been found to reduce inflammatory infiltration in local or systemic tissues, but the inflammation can’t be reduced as much as the BM-MSCs do when IP injection (Kagia et al. 2019).

## Conclusion and perspectives

Stem cell therapy is a novel approach for treating NEC. Whereas most research is still limited to animal experiments, studies on its long-term outcomes are lacking. Various challenges have hindered the translation of preclinical studies to clinical applications, including the safety of stem cell transplantation in infants. Hence, the exact mechanisms through which stem cells exert beneficial effects in NEC and the pathogenesis of NEC need to be studied in greater detail to facilitate successful clinical trials. Stem cell derivatives or conjunctive treatments with other activators may also have applications in the treatment of NEC in the future.

## Data Availability

Not applicable.

## Abbreviations

NEC: Necrotizing enterocolitis
ESCs: Embryonic stem cells
NSCs: Neural stem cells
ASCs: Adult stem cells
iPSCs: Induced pluripotent stem cells
HSCs: Hematopoietic stem cells
MSCs: Mesenchymal stem cells
ISCs: Intestinal stem cells
LGR5: Leucine-rich repeat-containing G protein-coupled receptor 5
IECs: Intestinal epithelial cells
IL: Interleukin
TGF-β: Transforming growth factor-β
BM-MSCs: Bone marrow-derived mesenchymal stem cells
IP: Intraperitoneal
AF-MSCs: Amniotic fluid-derived mesenchymal stem cells
COX-2: Cyclooxygenase 2
UC-MSCs: Umbilical cord-derived stem cells
P-MSCs: Placental-derived mesenchymal stem cells
ENS: Enteric neural system
E-NSCs: Enteric NSCs
AF-NSCs: NSCs isolated from the amniotic fluid
IV: Intravenous
EVs: Extracellular vesicles
MSC-CM: Conditioned medium from mesenchymal stem cell
HLA: Human leukocyte antigen
